# Farnesoid X receptor via Notch1 directs asymmetric cell division of Sox9^+^ cells to prevent the development of liver cancer in a mouse model

**DOI:** 10.1186/s13287-021-02298-6

**Published:** 2021-04-12

**Authors:** Mi Chen, Chenxia Lu, Hanwen Lu, Junyi Zhang, Dan Qin, Shenghui Liu, Xiaodong Li, Lisheng Zhang

**Affiliations:** 1 College of Veterinary Medicine/College of Biomedicine and Health, Huazhong Agricultural University, Wuhan, 430070 China; 2 The Clinical Medical College of Traditional Chinese Medicine, Hubei University of Chinese Medicine, Wuhan, 430065 China; 3 Hubei Provincial Hospital of TCM, Hubei Provincial Academy of TCM, Wuhan, 430061 China

**Keywords:** FXR, Symmetric cell division, Sox9, Liver cancer stem cell, Notch1

## Abstract

**Background:**

Asymmetrical cell division (ACD) maintains the proper number of stem cells to ensure self-renewal. The rate of symmetric division increases as more cancer stem cells (CSCs) become malignant; however, the signaling pathway network involved in CSC division remains elusive. FXR (Farnesoid X receptor), a ligand-activated transcription factor, has several anti-tumor effects and has been shown to target CSCs. Here, we aimed at evaluating the role of FXR in the regulation of the cell division of CSCs.

**Methods:**

The FXR target gene and downstream molecular mechanisms were confirmed by qRT-PCR, Western blot, luciferase reporter assay, EMAS, Chip, and IF analyses. Pulse-chase BrdU labeling and paired-cell experiments were used to detect the cell division of liver CSCs. Gain- and loss-of-function experiments in Huh7 cells and mouse models were performed to support findings and elucidate the function and underlying mechanisms of FXR-Notch1 in liver CSC division.

**Results:**

We demonstrated that activation of Notch1 was significantly elevated in the livers of hepatocellular carcinoma (HCC) in Farnesoid X receptor-knockout (FXR-KO) mice and that FXR expression negatively correlated with Notch1 level during chronic liver injury. Activation of FXR induced the asymmetric divisions of Sox9^+^ liver CSCs and ameliorated liver injury. Mechanistically, FXR directs Sox9^+^ liver CSCs from symmetry to asymmetry via inhibition of Notch1 expression and activity. Deletion of FXR signaling or over-expression of Notch1 greatly increased Notch1 expression and activity along with ACD reduction. FXR inhibited Notch1 expression by directly binding to its promoter FXRE. FXR also positively regulated Numb expression, contributing to a feedback circuit, which decreased Notch1 activity and directed ACD.

**Conclusion:**

Our findings suggest that FXR represses Notch1 expression and directs ACD of Sox9^+^ cells to prevent the development of liver cancer.

**Supplementary Information:**

The online version contains supplementary material available at 10.1186/s13287-021-02298-6.

## Background

Hepatocellular carcinoma (HCC) is the most common primary malignancy in the liver [1]. Cellular heterogeneity is a typical feature of HCC [2]. Previous study has suggested that tumor heterogeneity is partly attributed to the existence of a subgroup of cells with stem cell character, the so-called cancer stem cells (CSCs) [3]. These CSCs within tumor tissue exhibit the ability to self-renew and differentiate, resulting in new tumor [4]. Moreover, a lot of defined surface markers can be used to identify and isolate the liver CSCs from HCC, including CD133, Sox9, CD13, CD90, CD24, CD44, and so on [5–8].

CSCs, the tumor-initiating cells, possess the ability to self-renew through symmetrical or asymmetrical divisions [9]. In particular, CSCs tend to lose the ability to regulate the normal balance of symmetric and asymmetric divisions, resulting in the overgrowth of symmetrical division tumor cells [10, 11]. Previous studies have revealed different CSC division regulation modes, and the most notable of them is the Notch signaling pathway [12–15]. Four different Notch receptors in mammals have been discovered [16]. Previous reports revealed that inhibition of Notch in CSCs could reduce tumorigenicity and SCD [11, 16, 17]. Recent studies have suggested that Notch1 serves as the marker for oncogene and symmetric division [17–19]. In addition, constitutive activation of Notch1 intracellular domain (NICD1) in the mouse liver led to spontaneous HCC [20]. Another study has shown that activation of Notch1 increases the liver CSC marker Sox9 expression [20].

Sox9 plays a major role in cell differentiation, sex determination, and tumorigenesis. Previous study has shown that upregulation of sox9 promotes tumorigenicity in liver CSCs, while inhibition of Sox9 expression in liver CSCs reduces liver CSC tumorigenicity and SCD [11]. Furthermore, our previous results have shown that miR-126 promotes the differentiation of Sox9^+^ liver progenitor cells into hepatocytes, thus contributing to hepatic repair [21]. Taken together, these findings suggested that inhibition of Sox9^+^ cell symmetrical self-renewal could reduce liver tumorigenicity and injury.

FXR plays a critical role in regulating bile acid synthesis, lipoprotein metabolism, glucose metabolism, and liver regeneration [22–24]. Strong evidence has shown that a role for FXR in liver tumorigenesis, with expression levels inversely correlating with HCC progression and malignancy [25, 26]. Interestingly, FXR-knockout (FXR-KO) mice developed spontaneous HCC at the age of 12 months [27], which is consistent with the time to develop HCC in mice with constitutive activation of Notch1 reported in another previous study [20]. Notably, a recent study has revealed that FXR restricted CSC expansion and the Notch1 expression was elevated in lgr5^+^ CSCs isolated from FXR deficient mice [28]. Although links between FXR, Notch1, and CSCs have been suggested, the underlying mechanisms remain unclear. Therefore, we aimed to determine whether FXR could repress Notch1 expression and direct liver CSC asymmetrical division to prevent the development of liver cancer.

## Materials and methods

### Animals

FXR-KO mice (C57BL/6 J background) were provided by Jackson Laboratory. C57BL/6 J SPF mice were purchased from the Huazhong Agricultural University Experimental Animal Center. All animal procedures were approved by the animal ethics and welfare committee of Huazhong Agricultural University.

### Reagents and antibodies

CDCA, GW4064, and BrdU were purchased from Sigma Chemicals (CA, USA). Luciferase assay system was purchased from Promega (WI, USA). EMSA assay kit and ChIP assay kit were purchased from Beyotime (Shanghai, China). Antibodies against FXR (sc-13063X), Numb (sc-136554), rabbit immunoglobulin G-horseradish peroxidase (IgG-HRP) (sc-2004), and goat anti-mouse IgG-HRP (sc-2005) were purchased from Santa Cruz (CA, USA). Antibody against Notch1 (#4380) and NICD1 (#4147) was purchased from Cell Signaling Technology (MA, USA). CD133 (ab19898) antibody was purchased from Abcam (MA, USA). Sox9 (AB5535) and BrdU (MAB3424) antibodies were purchased from Millipore (MA, USA). CD133-phycoerythrin (372803) antibody was purchased from BD Biosciences (CA, USA). Antibody against alpha-Tubulin (66031-1-Ig), LaminB (66095-1-Ig) was purchased from Proteintech (Wuhan, China). GAPDH antibody (BM-1623) was purchased from the Boster Biological Technology (Wuhan, China).

### Cell culture and siRNA transfection

Huh7 was purchased from the Cell Bank of the Chinese Academy of Sciences (Shanghai, China). The cell lines were cultured in DMEM supplemented with 10% fetal bovine serum (FBS) and 1% streptomycin and penicillin under standard culture conditions. The small interfering RNA (siRNA) oligonucleotides for FXR (Si-FXR) and the negative control (Si-NC) were designed and synthesized by the Shanghai GenePharma (Shanghai, China). The sequences were as follows: FXR siRNA: Sense: 5′-GAGGAUGCCUCAGGAAAUATT-3′, anti-sense: 5′-UAUUUCCUGAGGCAUCCUCTT-3′. Transfection of siRNAs into Huh7 cells was accomplished by RNAimax (Invitrogen, Carlsbad, USA) according to the manufacturer’s instructions. The cells were collected at 36 h or 48 h post transfection. The siRNA-FXR and negative control (Si-NC) sequences were listed in Table S1.

### Animal treatment

To confirm the negative correlation between FXR and Notch1, 8-week-old WT mice were fed with standard chow containing 0.1% of 3,5 diethoxicarbonyl-1,4 dihydrocollidine (DDC) and normal drinking water for 1 week. Untreated control mice were fed with a standard rodent chow diet. For CCl_4_ induced liver injury, mice received a twice-weekly intraperitoneal (IP) injection of CCl_4,_ twice per week for 2 weeks. Carbon tetrachloride was mixed with Paraffin oil at a ratio 1:4. The control group was treated with an equal amount of vehicle.

To determine whether FXR activation inhibits Notch1 in vivo, 8 week-old WT and FXR-KO mice were fed with standard chow containing 0.1% DDC for 1 week or received a twice-weekly IP injection of CCl_4,_ twice per week for 2 weeks. In addition to the DDC and CCl_4_-treatment, 8 week-old WT and FXR-KO mice were orally gavaged with either control (4:1 of PEG-400 and Tween 80) or GW4064 (50 mg/kg body weight) once every 2 days for 1 week or 2 weeks. For BrdU staining, mice were injected intraperitoneally of BrdU at a dose of 50 mg/kg body weight every 2 h, repeated 4 times before tissue collection. At the end of the study, mice were terminated by using cervical dislocation.

### Serum transaminase levels and liver histopathological examination analysis

The AST and ALT levels were measured by assay kits (C010-2, C009-2) purchased from Nanjing Jiancheng (Nanjing, China). For the histologic assessment, the livers were fixed in 4% formaldehyde for 24 h and embedded in paraffin. Liver sections (5 μm) were deparaffinized and fixed. In all experimental groups, the 5-μm-thick sections of the formalin-fixed and paraffin-embedded livers were processed for hematoxylin and eosin staining (H&E) to estimate the degree of hepatic lesions.

### Quantitative real-time PCR

RNA was extracted using RNAiso plus (TaKaRa, Japan), and the cDNA synthesis was conducted using a cDNA synthesis kit (TOYOBO, Japan). Quantitative real-time PCR (qRT-PCR) was performed using the SYBR GREEN qPCR mix (TOYOBO, Japan) according to the manufacturer’s instructions. The data were detected by using the ABI CFX Connect TM Real-Time PCR Detection System (ABI, USA). The ^ΔΔ^CT method was calculated to obtain the fold expression levels. The primer pairs for quantitative real-time PCR were listed in Table S2.

### Western blotting

Liver tissues and cells were lysed with lysis buffer (Beyotime, Jiangsu, China) to obtain whole-protein extracts. Nuclear extracts were prepared according to the instructions of the nuclear and cytoplasmic extraction kit (BestBio, Shanghai, China). The protein samples (20 mg) were separated via 10% SDS-PAGE and then were transferred onto polyvinylidene fluoride (PVDF) membranes (Millipore, USA). After being blocked with 5% skimmed milk in Tris-buffered saline/Tween-20 (TBST), the membranes were incubated with the primary antibodies overnight at 4 °C and then incubated with the respective HRP-conjugated secondary antibodies for 1.5 h. Finally, the membranes were visualized with enhanced chemiluminescence (ECL) (Bio-Rad, USA). Quantitative analysis of Western blot assays was carried out with ImageJ software.

### Immunofluorescence

For FXR and Notch1 staining, frozen tissues were embedded in Tissue Tek O.C.T. compound (Sarura Finetek, CA) and 10 μm using a Leica CM3050S Cryostat (Leica, Heerbrugg, Switzerland). Frozen sections were stained with anti-FXR (1:100) and anti-Notch1 (1:200). After being counterstained with DAPI (Invitrogen), the slides were observed under a fluorescent microscope.

Pulse-chase BrdU labeling and paired-cell assays were used to investigate the cell division of liver CSCs. The DNA of parental cells was prelabeled with BrdU (1 μM) for 2 weeks. Subsequently, CD133-positive cells were enriched by fluorescence-activated cell sorting (FACS), scattered into single cells for paired-cell formation. Afterwards, the single cells were seeded on coated coverslips in a normal medium (DMEM + 10% FBS) containing GW4064 (10 μM) for the experiment group or DMSO for the control group. After a 24-h culture, the paired cells were fixed and permeabilized. Then the resultant cells were sequentially immersed first in 1 N HCl and then in 2 N HCl to open the DNA structure. Immediately after the acid washes, cells were buffered in borate buffer (0.1 M, pH 8) at room temperature. And then, they were washed and incubated overnight with antibodies specific for BrdU or the stem cell markers. The results were observed under a fluorescent microscope. Any ambiguous segregation of BrdU was excluded from the analysis.

For further analysis of Sox9^+^ liver CSC analysis in vivo, the livers from WT and FXR-KO mice were fixed in 4% formaldehyde-PBS solution, embedded in paraffin, and sectioned at 10 μm, and paraffin sections were then prepared and stained with anti-Sox9 (1:100; Millipore), anti-BrdU(1:100; Millipore), and anti-α-tubulin (1:200; Proteintech) antibodies. After being counterstained with DAPI (1:1000; Invitrogen), the slides were observed under a fluorescent microscope.

We evaluate symmetric and asymmetric percentages based on the fluorescence signal intensity of each cell acquired by a fluorescent microscope and quantified by ImageJ. Thresholds to determine BrdU ^high/low^ asymmetric were set for experimental replicates. Briefly, if the fluorescent intensity of BrdU in the BrdU^high^ daughter cell was more than 2-fold higher than that in BrdU^−/low^ daughter cell, we defined this cell division mode as ACD. If the BrdU intensity less than 2-fold difference in the daughter pairs, we defined this cell division mode as SCD. For immunofluorescence (IF) staining assay, dividing liver cancer cells were fixed and costained with BrdU/Notch1 antibodies, we calculated and analyzed the fluorescent intensities of BrdU and Notch1 within one daughter cell. If the fluorescent intensity difference between the two proteins was more than 2-fold, we defined this expression pattern as inverse expression. If the fluorescent intensity difference was less than 2-fold, we defined this expression pattern as co-expression [9, 11].

### Plasmid construction, transfection, and luciferase reporter assay

The Notch1 intracellular domain sequence was determined to be 5214–7686 bp. Notch1 intracellular domain cDNA was obtained by reverse transcription from PolyA mRNA purified from LO2 cells, the primer sequences were listed in Table S2. The cDNA was amplified and subcloned into the pcDNA3.1 expression vector (Invitrogen), which was designated OE-Notch1. The purified OE-Notch1 (pcDNA3.1-Notch1) and vector (pcDNA3.1) plasmids were transfected into cells using Lipofectamine 2000 (Invitrogen, Carlsbad, USA) according to the manufacturer’s instructions.

Putative FXRE/DR7 in the Notch1 promoter region was predicted using an online algorithm (NUBI Scan: http://www.nubiscan.unibas.ch/). Based on this prediction (Fig. 5a), LO2 cell genomic DNA was used to clone Notch1 promoter region into the pGL3-basic vector (Promega, USA) with the Notch1 promotor, and the resultant plasmids with the Notch1 promoter region were named as follows: pGL3-Notch1 FXRE-wt (− 1700 to + 10) (also named pGL3-DR7-wt) and pGL3-Notch1 FXRE-mut, derived from pGL3-Notch1 FXRE-wt, containing mutations in the FXRE/DR7 element (TGACCCcaagatgTAACCC; with the mutated bases underlined). PGL3-Notch1 FXRE-wt plasmid or pGL3-Notch1 FXRE-mut was separately co-transfected with the Renilla luciferase expression vector pRL-TK (Promega, USA) into HepG2 cells by Lipofectamine 2000 (Invitrogen, Carlsbad, USA). After 6-h co-transfection, the resultant HepG2 cells were treated with GW4064 (10 μM) for 36 h. These cells were lysed using the dual-luciferase assay kit (Promega, USA). The luciferase activity was measured by a Fluoroskan Ascent FL (Thermo Scientific, USA). Firefly luciferase activity was normalized into that of Renilla luciferase activity.

### Electrophoretic mobility shift assay

EMSA was performed to evaluate the interaction of FXR protein with DR7 element. Nuclear protein was prepared from GW4064-treated HepG2 cells using the Active Motif Nuclear Extract Kit (Active Motif, CA, USA, nos. 40010 and 40410). The DR7 element interaction of FXR was detected by EMSA Kit, and the DNA-binding reaction system and double-stranded oligonucleotides were listed in Table S4 and Fig. 5c, respectively. For supershift assays, the nuclear protein was pre-incubated with antibodies against FXR (Santa Cruz, CA, USA, sc-13063X) for 20 min before the addition of the DR7 probe in the reaction buffer at 25 °C for 20 min. The reactions were analyzed by electrophoresis in a non-denaturing 6.6% polyacrylamide gel, followed by development.

### ChIP

ChIP assay for HepG2 cells GW4064-treated was performed according to the manufacturer’s instructions a ChIP Assay kit (Beyotime, Jiangsu, China). The anti-FXR antibody and IgG were used in the immunoprecipitations. The ChIP-isolated DNA was subjected to PCR amplification using the primer pair spanning the FXRE/DR7 in Notch1 promoter region (the primer sequences are listed in Table S2).

### Statistical analysis

Statistical analyses were conducted with GraphPad Prism software 6.01. All data were presented as the mean ± SEM. of at least three separate experiments. Normal distribution of all variables was tested, and if all variables met normal distribution, statistically significant differences were assessed by the two-tailed Student’s *t* test or by one-way ANOVA tests; otherwise, the Kruskal-Wallis test was used. Statistical significance was set at *P* < 0.05 (*), *P* < 0.01 (**), and *P* < 0.001 (***). More methods and materials can be found in the supplemental information.

## Results

### Notch1 level is elevated in the livers of FXR-KO mice and inversely correlate with FXR level in chronic liver injury

**Fig. 1:**
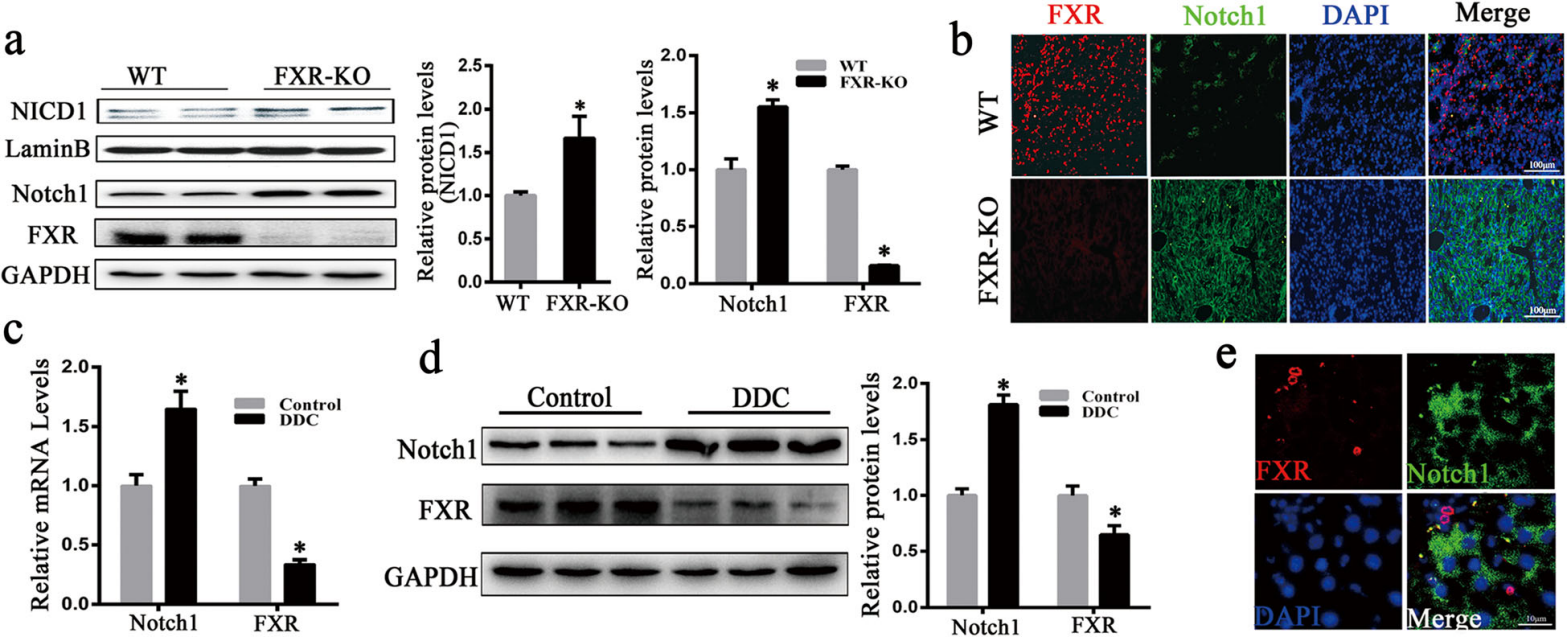
Notch1 level is elevated in the livers of FXR-KO mice and inversely correlate with FXR level in the DDC-treated mouse livers. **a** Expression of NICD1 and Notch1 was elevated in liver tumor samples from 12-month-old FXR-KO mice, normalized to LaminB or GAPDH. **b** Representative photomicrographs shown expression of FXR and Notch1 in 12-month-old WT and FXR-KO mouse liver tissues as assessed by an IF assay. Scale bars: 100 μm. **c** Expression of FXR and Notch1 mRNAs in the livers of WT and DDC-treated mice. **d** Expression of FXR and Notch1 in WT and DDC-treated mice was examined by western blotting, normalized to GAPDH. **e** The liver of DDC-treated mouse samples was co-stained with anti-FXR and anti-Notch1 antibodies, then counterstained with DAPI for confocal microscopy. Scale bars: 10 μm. Data were presented as mean ± SEM of three independent experiments. *p < 0.05

FXR-KO mice spontaneously developed HCC when they aged [27], but the corresponding mechanisms are still not completely understood. The activation of Notch1 was found to be one of the key events in the development of liver cancer [20]. In this study, we tested whether the pathway was activated in the livers of FXR-KO mice. Figure S1 showed the liver of 12-month-old FXR-KO mice with tumors. As expected, the result indicated that the levels of the nuclear translocation of NICD1 were elevated in the FXR-KO mice (Fig. 1a). Moreover, the protein levels of Notch1 were significantly increased in the FXR-KO mice (Fig. 1a). Intense Notch1 staining was observed in IF analysis of FXR-KO mouse liver, while no intense Notch1 staining was observed in wild-type (WT) mice (Fig. 1b). High expression of FXR and low Notch1 expression were observed in the 12 month-old WT mice (Fig. 1b). A previous study has shown that most HCC develops in a chronically injured liver [29]. To determine the inverse relationship between FXR and Notch1, we detected the FXR and Notch1 levels in the DDC and CCl_4_ mouse model. As shown in Fig. 1c, d, the expression of FXR was substantially decreased in the liver of DDC-fed mice, while the expression of Notch1 was significantly increased. Similar results were obtained from the mice treated with CCl_4_ (Figure S2). In addition, we detected the expression of FXR and Notch1 expression in DDC-treated mice by IF, and it was shown that FXR was not co-expressed with Notch1, further confirming the FXR inversely correlated with Notch1 (Fig. 1e). Taken together, these data suggest FXR inhibits Notch1 expression and activity.

### FXR inhibits Notch1 expression in HCC cells and induces the asymmetric division of liver CSCs

**Fig. 2:**
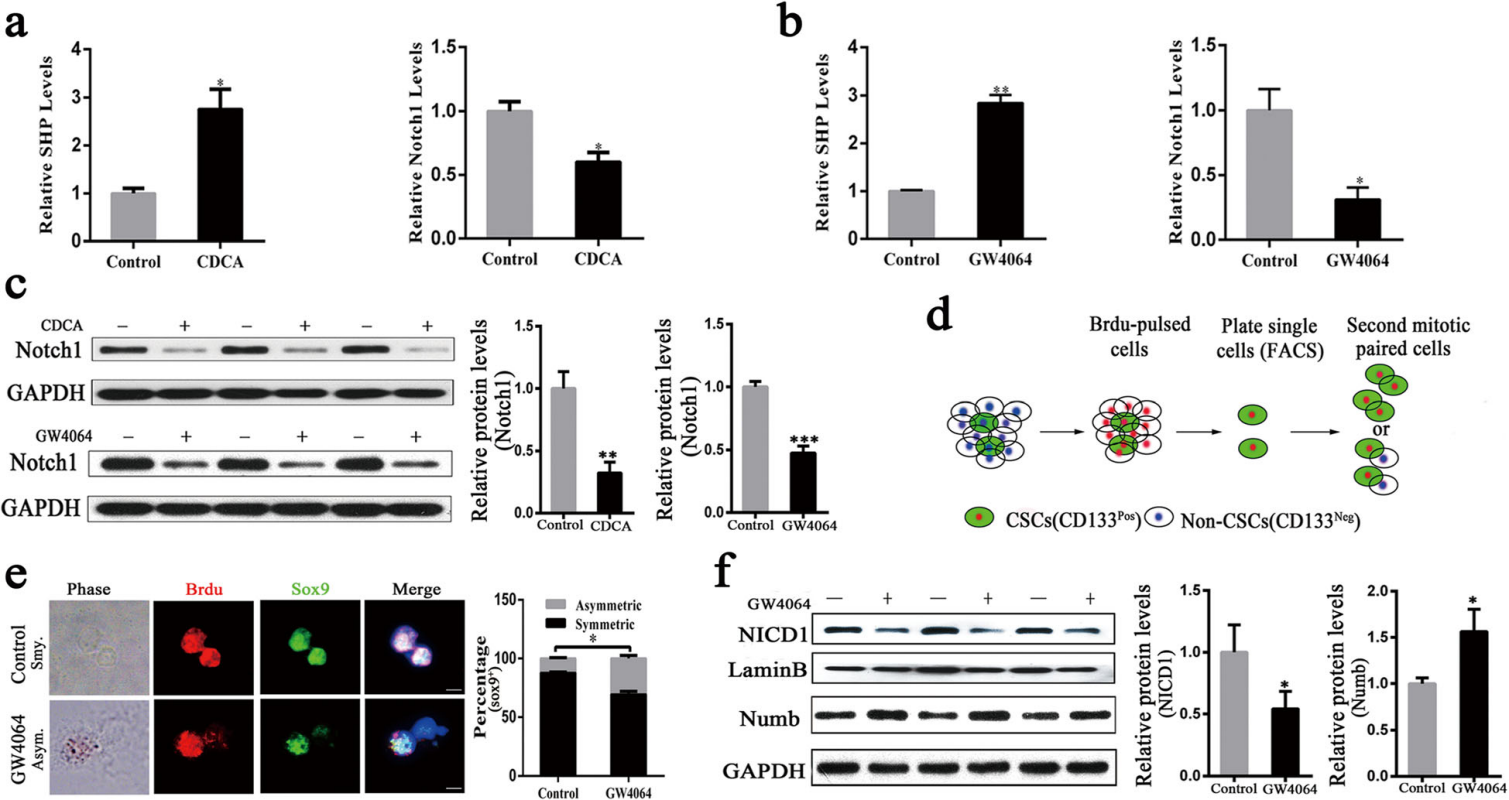
FXR inhibits Notch1 expression in HCC cells and directs the asymmetric division of Sox9^+^ liver CSCs. **a** Huh7 cells were treated with CDCA (100 μM) or DMSO for 24 h, and the expression of SHP and Notch1 was examined by quantitative real-time PCR. **b** Huh7 cells were treated with GW4064 (10 μM) or DMSO for 24 h, and the expression of SHP and Notch1 was assayed by quantitative real-time PCR. GAPDH was used as an internal control for the examination of SHP and Notch1. **c** Effects of FXR agonists (CDCA or GW4064) on Notch1 protein expression. Huh7 cells were treated with CDCA (100 μM) or GW4064 (10 μM) for 36 h. Total protein samples were collected and subjected to Western blotting analysis to detect Notch1 protein expression, normalized to GAPDH. **d** the schematic depiction shown the BrdU pulse-chase and paired-cell assays. **e** Left: Representative images of the paired-cell assay in CD133^+^ liver CSCs from Huh7 cells. BrdU, red; DNA, blue; Sox9, green. Scale bars: 5 μm. Right: Quantification of BrdU asymmetry or symmetry in Sox9^+^ liver CSCs maintaining in 10% stripped FBS-containing medium supplemented with GW4064 (10 μM) or DMSO for 24 h. **f** Western blotting was used to detect the protein levels of NICD1 and Numb in Huh7 cells maintaining in 10% stripped FBS-containing medium supplemented with GW4064 (10 μM) or DMSO for 36 h. Data were presented as mean ± SEM of three independent experiments. *p < 0.05, **p < 0.01, ***p < 0.001

Nuclear receptors (NRs) are ligand-activated transcription factors regulating a lot of target genes [30]. FXR as the nuclear receptor requires specific ligand activation to exert its effects. To investigate whether FXR inhibits Notch1 expression, Huh7 cells were treated with the FXR natural ligand chenodexycholic acid (CDCA), followed by quantitative real-time PCR to detect the expression of Notch1. Previous studies have reported that the increased mRNA levels of a small heterodimer partner (SHP) demonstrated the activation of FXR [31, 32]. Based on it, the mRNA levels of the SHP were measured as positive controls in this study. The results revealed a significant upregulation of SHP expression, suggesting that FXR was activated (Fig. 2a). Figure 2a showed that Notch1 expression was inhibited after FXR activation. The treatment with GW4064, another synthetic highly specific FXR agonist, led to Notch1 expression suppression (Fig. 2b). A similar suppression effect on Notch1 protein levels was also observed. Figure 2c illustrated that CDCA or GW4064-treated Huh7 cells exhibited lower Notch1 protein levels than DMSO-treated cells of the control group. These data further indicated that FXR activation repressed Notch1 expression.

As a liver CSC marker, Sox9 has been reported to have the ability to facilitate cancer cell growth and participate in the symmetric division and asymmetric division of CSCs [6, 11]. The inhibition of Notch in liver CSCs was found to promote asymmetric division [11]. Our study found that the activation of FXR decreased the expression of Notch1 in Huh7 cells (Fig. 2c). Subsequently, we examined whether FXR influenced the cell division of liver CSCs through pulse-chase BrdU labeling and paired-cell experiments. The results have shown that almost all cells were labeled by Brdu (Figure S3). The newly synthesized DNA was assigned to the differentiated daughter cells, and the prelabeled DNA was distributed to the daughter CSC [33, 34]. The BrdU pulse-chase experiment model was shown in Fig. 2d. Using this model, liver CSCs were further sorted by fluorescence-activated sorting CD133^+^ cells. The results showed that the asymmetric division frequency of Sox9^+^ liver CSCs was significantly increased under the stimulation of GW4064, compared with that of DMSO-treated cells of the control group (Fig. 2e). Consistently, we found that the nucleus translocation of NICD1 was decreased after FXR activation (Fig. 2f). Numb is a cell fate determinant for many kinds of CSCs and has been used as a marker for distinguishing symmetric versus asymmetric division [11]. Previous study has shown that Numb promotes the ubiquitination of Notch1 receptor and the degradation of Notch1 intracellular domain [35]. Western blotting showed the increased expression of Numb after FXR activation (Fig. 2f), which further suggested that pharmacological activation of FXR played a vital role in promoting the asymmetric division of liver CSCs.

### FXR promotes asymmetric division of liver CSCs through regulation of Notch1

**Fig. 3:**
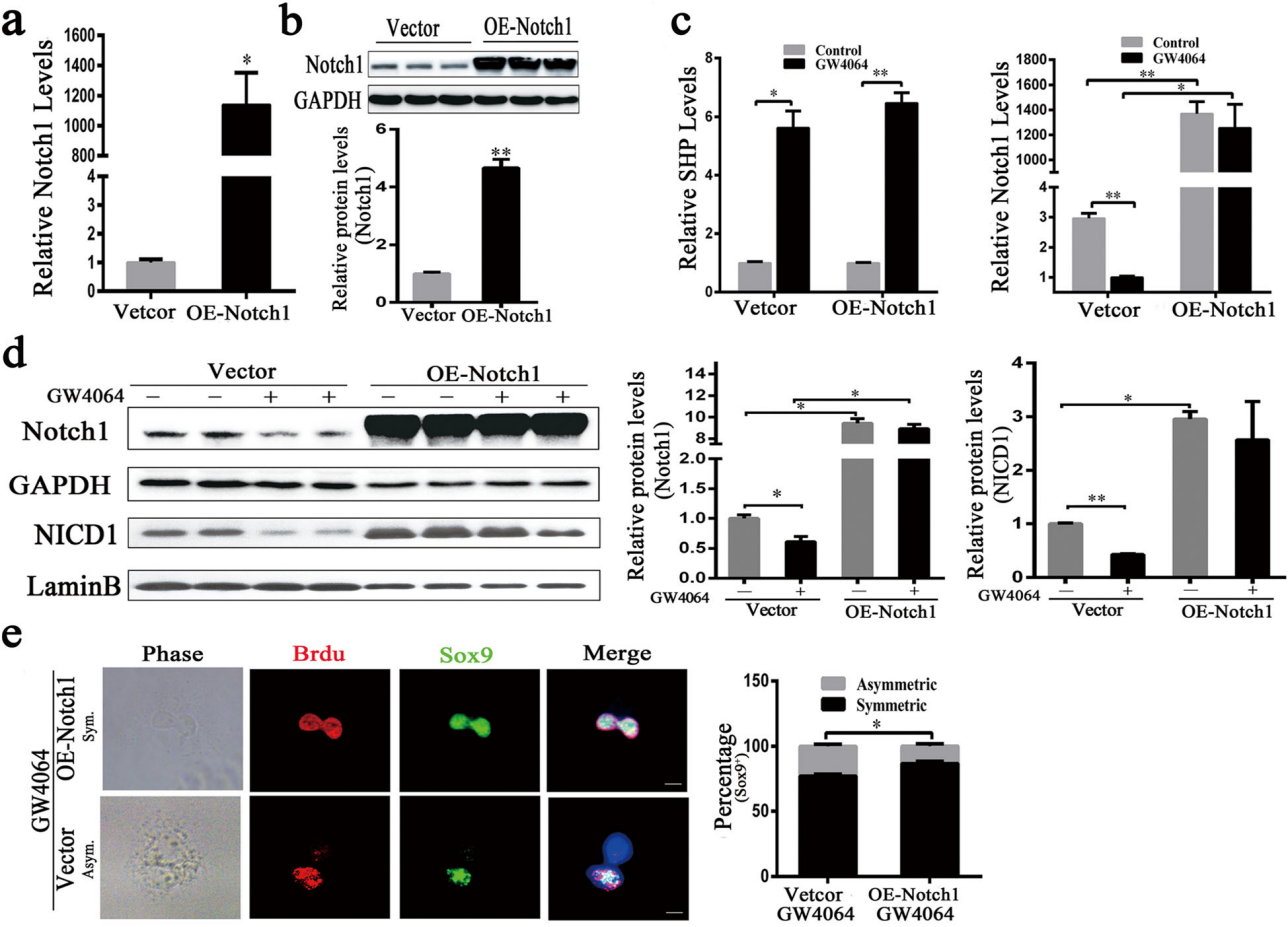
The overexpression of Notch1 reverses the FXR agonist-induced asymmetric cell division in liver CSCs. **a** Huh7 cells were transfected with Notch1 expression plasmid or vector plasmid, and quantitative real-time PCR confirmed Notch1 overexpression. **b** Huh7 cells were transfected with Notch1 expression plasmid or vector plasmid, Western blottings confirmed Notch1 overexpression, normalized to GAPDH. **c** After transfected with Notch1 expression plasmid or vector plasmid for 7 h, Huh7 cells were treated with GW4064 (10 μM) for another 24 h, and quantitative real-time PCR was assayed for SHP and Notch1 expression. **d** After transfected with Notch1 expression plasmid or vector plasmid for 6 h, Huh7 cells were treated with GW4064 (10 μM) for another 36 h, and Western blotting was assayed for Notch1 and NICD1 expression, normalized to LaminB or GAPDH. **e** Left: Representative images of the paired-cell assay of liver CSCs from Huh7 cells. BrdU, red; DNA, blue; Sox9, green. Scale bars: 5 μm. Right: Quantification of BrdU asymmetry or symmetry in liver CSCs after Notch1 overexpression expression maintained treated with GW4064 (10 μM) medium. Data were means of three separated experiments ± SEM, *p < 0.05, **p < 0.01

We further examined whether ACD regulation by FXR was mediated through Notch1 or not. First, we transfected with Notch1 overexpression plasmid or vector plasmid into Huh7 cells, the overexpression of Notch1 was confirmed by real-time quantitative PCR and western blot (Fig. 3a, b). The results showed that the effect of Notch1 suppression by GW4064 was partially negated by overexpression of Notch1, which was evident by the significantly higher Notch1 expression and translocation of NICD1 into nucleus levels (Fig. 3c, d). In addition, we transfected with Notch1 overexpression plasmid or vector plasmid into the sorted CD133^+^ liver CSCs. We found that overexpression of Notch1 also increased Sox9^+^ liver CSCs symmetric division in GW4064-containing medium (Fig. 3e).

**Fig. 4:**
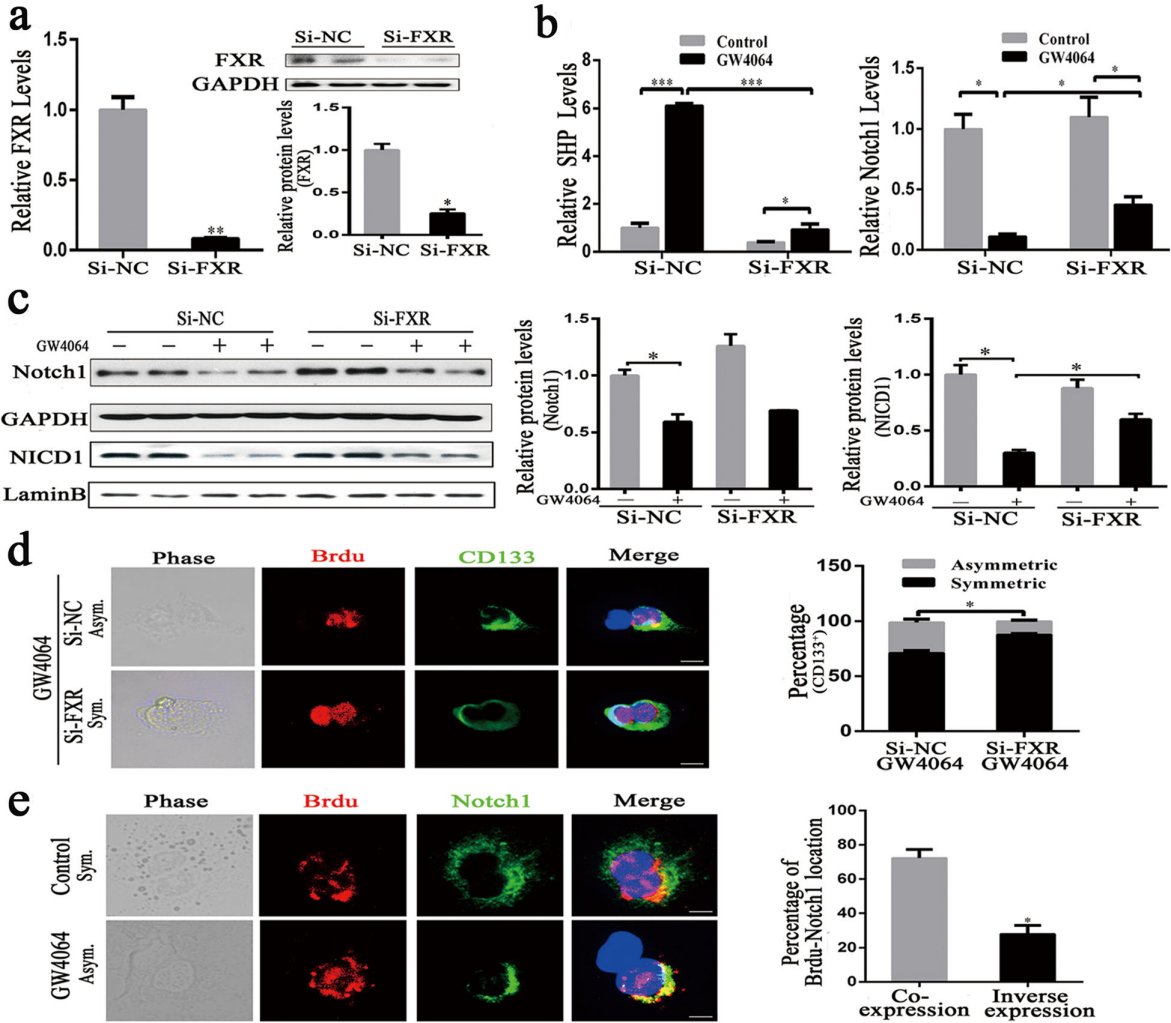
FXR promotes asymmetric division of liver CSCs through regulation of Notch1. **a** The expression of FXR was examined by quantitative real-time PCR and Western blotting, normalized to GAPDH. **b** After interfering FXR expression for 12 h, Huh7 cells were treated with GW4064 (10 μM) or control DMSO for another 24 h, and quantitative real-time PCR was assayed for SHP and Notch1 expression. **c** After interfering FXR expression for 12 h, Huh7 cells were treated with GW4064 (10 μM) or Control DMSO for another 36 h, and Western blotting was assayed for Notch1 and NICD1 expression, normalized to LaminB or GAPDH. **d** Left: Representative images of the paired-cell assay of liver CSCs from Huh7 cells. BrdU, red; DNA, blue; CD133, green. Scale bars: 5 μm. Right: Quantification of BrdU asymmetry or symmetry in liver CSCs after interfering FXR expression maintained treated with GW4064 (10 μM) medium. **e** Left: Representative images of the paired-cell assay of liver CSCs from Huh7 cells. BrdU, red; DNA, blue; Notch1, Green. Scale bars: 5 μm. Right: The percentage of BrdU and Notch1 co-expression or inverse expression in daughter cells of CD133^+^ liver CSCs from Huh7 cells undergoing division. Data were means of three separated experiments ± SEM, *p < 0.05, **p < 0.01, ***p < 0.001

Next, we knocked-down the FXR expression by siRNA silencing experiment. As shown in Fig. 4a, FXR-siRNA significantly reduced the levels of FXR and that GW4064-mediated FXR activation was neutralized in the presence of the FXR-siRNA (Fig. 4b). FXR knockdown eliminated the Notch1 expression suppression and translocation of NICD1 into the nucleus by GW4064 (Fig. 4c), which further supports the direct contribution of FXR to ACD. To assess the role of FXR in regulating the asymmetric division of liver CSCs, we depleted FXR in the sorted CD133^+^ liver CSCs by siRNAs. As expected, FXR knockdown decreased the GW4064-mediated ACD (Fig. 4d). We also found that Notch1 was highly colocalized with BrdU (Fig. 4e). These findings suggest that FXR induces asymmetric division of liver CSCs through inhibiting the Notch1 signaling pathway.

### FXR activation reduces the transcriptional activity of the Notch1

**Fig. 5:**
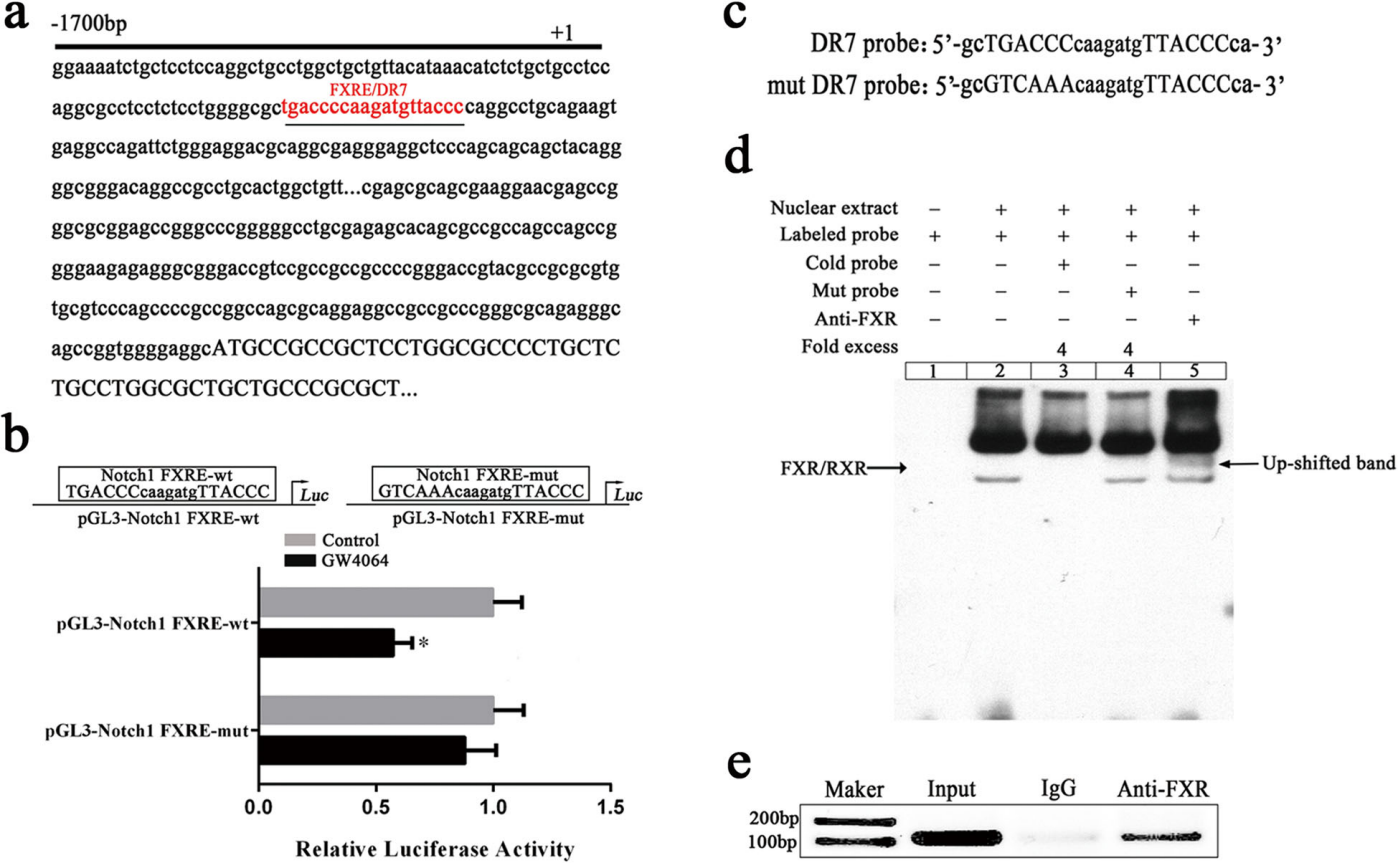
FXR binds to FXRE in Notch1 promoter region and suppresses Notch1 transcriptional activity. **a** Potential FXRE/DR7 in the Notch1 promoter region was predicted that the site of Notch1 was indicated by red letter, and the FXRE/DR7 was underlined. **b** pGL3-Notch1 FXRE-wt and pGL3-Notch1 FXRE-mut were separately co-transfected with the Renilla luciferase expression vector pRL-TK into HepG2 cells. After 6 h of incubation, the cells were treated with DMSO or GW4064 (10 μM) for 36 h. **c** The sequences of the DR7 probe and mutant DR7 probe were shown. **d** EMSA analysis of the binding of FXR proteins to the DR7 was performed. The position of the up-shifted FXR/RXR a complex was indicated. **e** ChIP assays were performed using chromatin isolated from GW4064-treated HepG2 cells. Data were means of three separated experiments ± SEM, *p < 0.05

FXR, ligand-dependent transcriptional regulator, is known to regulate the target gene expression by binding to FXRE. To clarify whether the Notch1 gene promoter region for potential FXRE, we used online algorithm (NUBIScan, http://www.nubiscan.unibas.ch/) to predict the Notch1 promoter region potential FXRE (Fig. 5a). Then, we constructed luciferase reporters pGL3-Notch1 FXRE-wt or mutated pGL3-Notch1 FXRE-mut. These constructs were co-transfected into HepG2 cells with the pRL-TK. Figure 5b showed that the GW4064-treated pGL3-Notch1 FXRE-wt group produced 2-fold decrease of luciferase activity in comparison with the DMSO-treated control group, indicating that this region may exist FXRE. In addition, the luciferase activity of the pGL3-Notch1 FXRE-mut group was unaffected after GW4064-treated (Fig. 5b). These results revealed that Notch1 may be a target gene of FXR.

Next, we used electrophoretic mobility shift assay (EMSA) to confirm the interaction of the DR7 element with FXR. The DR7 and mutant DR7 probes were shown in Fig. 5c. The result indicated that the interaction of the DR7 element with the nuclear extracts of HepG2 cells led to the production of DNA/protein shift band (Fig. 5d). Moreover, the binding was specific as it was specifically competed out by the unlabeled (cold) DR7 probe but not by the mutant DR7 probe (Fig. 5d). The supershift assay indicated that FXR protein was contained in the protein-DNA complex (Fig. 5d). As shown in Fig. 5e, ChIP assays were also performed to further verify FXR converges on the DR7. These data suggest that FXR inhibits Notch1 expression by binding to the FXRE/DR7.

### FXR directs Sox9^+^ cell asymmetric division in vivo

**Fig. 6:**
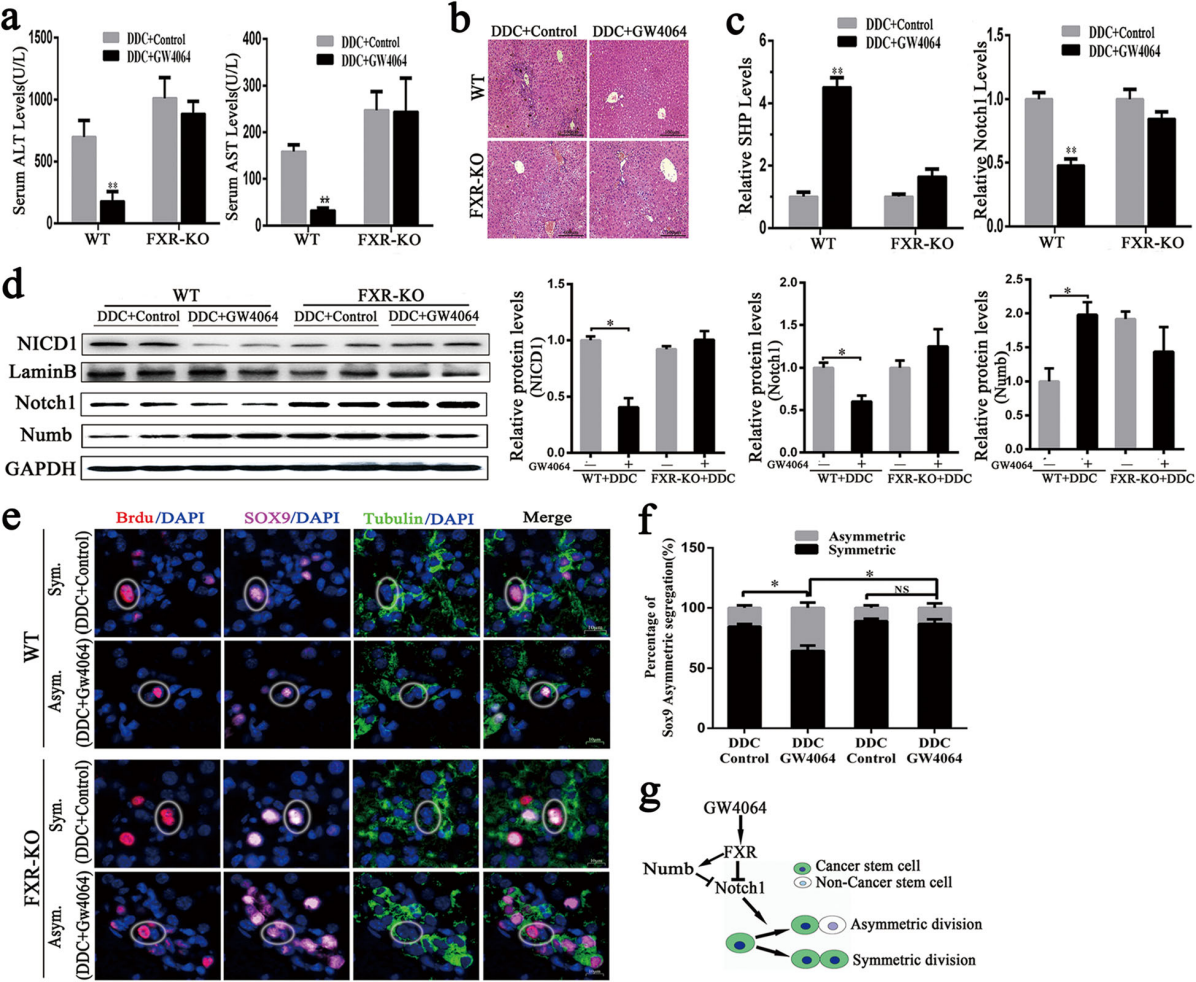
FXR activation inhibits Notch1 and promotes Sox9^+^ cells asymmetric division. **a** Serum ALT (left) and AST (right) activities were determined. **b** H&E staining of liver sections from WT and FXR-KO mice. Scale bars: 100 μm. **c** Liver tissues were subjected to quantitative real-time PCR assay for determination of SHP (left) and Notch1 (right) mRNA levels. **d** Liver tissues were applied to Western blotting analysis of NICD1, Notch1 and Numb protein levels, normalized to LaminB or GAPDH. **e**, **f** Representative images **e** and quantification **f** the asymmetric division frequency of Sox9^+^ cells in DDC fed WT and FXR-KO mice with either (DDC/control) or (DDC/GW4064) treatment. Scale bars: 10 μm. **g** A model for the regulation of asymmetric division by FXR-Notch1. Data represented the mean ± SEM (*N* = 4–6). Statistical significance of differences between each treatment and control group (*p < 0.05; **p < 0.01) were determined

In order to determine whether FXR activation could induce ACD of Sox9^+^ cells in vivo and to reveal its molecular mechanisms, WT and FXR-KO mice received DDC-diet. As expected, the treatment with GW4064 reduced DDC-induced liver injury, which could be attributed to a significant decline in the increase of serum ALT and AST levels (Fig. 6a). However, the protective effect of GW4064 against serum ALT and AST elevation were abolished in FXR-KO mice (Fig. 6a). Moreover, the histological evaluation revealed that GW4064 relieved DDC-induced liver injury in WT mice, but DDC-induced liver injury was not inhibited in FXR-KO mice (Fig. 6b). As mentioned earlier, DDC diet treatment resulted in upregulation of Notch1 expression in WT mice (Fig. 1c, d). After DDC treatment, FXR activation by GW4064 reduced Notch1 mRNA levels in WT mice but not in FXR-KO mice (Fig. 6c). Consistent with the change in mRNA levels, GW4064 treatment significantly decreased the protein levels of the nucleus translocation of NICD1 and Notch1 in WT but not in FXR-KO mice (Fig. 6d). Furthermore, we also examined Notch1 antagonist Numb expression and found that GW4064 treatment increased Numb protein levels in WT mice, but not in FXR-KO mice (Fig. 6d). We validated the relationship between FXR and Notch1 role in another chronic liver injury model which was induced by twice weekly CCl_4_ injections for 2 weeks and similar results were found in the CCl_4_ chronic liver model (Figure S4).

In order to confirm whether Sox9^+^ cell asymmetric division is triggered by FXR activation in vivo, Sox9^+^ cells were co-stained with tubulin to identify dividing liver CSCs pairs. Subsequently, BrdU incorporating Sox9^+^ cells and tubulin were concurrently stained to validate division symmetry. The results indicated that pharmacological activation of FXR increased the frequency of Sox9^+^ cell asymmetric division in WT mice but not in FXR-KO mice (Fig. 6e, f). Furthermore, the frequency of Sox9^+^ cell asymmetric division in FXR-KO mice was significantly decreased after GW4064 treatment compared to WT mice (Fig. 6e, f). This study reveals the mechanism by which FXR directed the liver CSC asymmetric division, as shown in Fig. 6g. Namely FXR activation inhibited Notch1 expression by direct FXRE binding to suppress the transactivity of Notch1. Meanwhile, FXR also positively regulated Numb expression, contributing to a feedback circuit, which decreased Notch1 activity and directed Sox9^+^ cells asymmetric division to prevent the development of liver cancer.

## Discussion

CSCs have been found in multiple cancers, containing the liver, lung, brain, breast, colon, and so on [36]. CSCs are different from normal stem cells, because they lose the ability to normally regulate their mode of cell division, which gives rise to a large quantity of CSC generation and tumor growth [37, 38]. Notch is a critical regulator of cell divisions in many types of CSCs [11, 18, 19]. In colon CSCs, the elevated Notch1 signaling inhibits asymmetric division of colon CSCs [19]. Our study demonstrated that FXR suppressed Notch1 expression and increased asymmetric division in liver CSCs, which is consistent with a previous study that the inhibition of Notch activity reduces symmetric division in liver CSCs [11]. Previous study has reported that Numb contributes β-catenin degradation to regulate colorectal CSC asymmetric division [39]. Our study revealed that FXR acted as an upstream regulator of Notch1 and Numb, thereby directing ACD. Recently, it is illustrated that FXR represses β-catenin activation and restricts colorectal CSCs Lgr5^+^ expansion [40]. Our findings provided the other model to illustrate the molecular mechanism by which FXR activation blocked CSC growth in cancer cells.

Metabolic regulation of stemness is increasingly recognized as fundamental in the control of CSCs fate [41]. Recently, studies have suggested that glycolysis contributes to the proliferation of CSCs, while glycolysis inhibition or glucose deprivation leads to a decline in the CSC number [42, 43]. Moreover, the metabolism of lipids and cholesterol is also an important factor in regulating CSCs proliferation [44]. Metabolic nuclear receptors as transcription factors respond to changes in metabolites. For example, nuclear receptor PPAR-δ has been identified to play a major role in stem cell fate determination and asymmetric division by regulating metabolic pathways [45]. Deletion PPAR-δ inhibits the ACD of hematopoietic stem cells [45]. Our study revealed that another metabolic nuclear receptor FXR as a cell fate determinant in HCC repressed Notch1 to enhance asymmetric division. FXR has been regarded as an important regulator of metabolism in the maintenance of lipids and glucose homeostasis [23]. And FXR has also been found to suppress tumors in liver tissue [24]. FXR-KO mice whose bile acids synthesis has been dysregulated develop hepatitis and liver cancer spontaneously [46]. Interestingly, this study revealed that Notch1 is significantly increased in livers of FXR-KO mice developing spontaneous HCC. Additionally, Notch1 also directs SCD and promotes the CSC phenotype and tumorigenicity [17, 19]. However, whether Notch1 could direct SCD during the spontaneous development of HCC in FXR-KO mice requires further investigation.

## Conclusion

In summary, our study reveals a critical role of FXR-Notch1 pathway in guiding the asymmetric division of liver CSC and preventing liver tumor development, suggesting that this pathway may be exploited for the targeted therapy of liver CSCs.

## Supplementary Information

**Additional file 1: Figure S1.** Representative photomicrographs of liver lesions from 12 month-old WT and FXR-KO mice. a. Liver tumors in 12-month-oldWT and FXR-KO mice. Circles show the tumor nodules. **Figure S2.** Hepatic FXR Levels Inversely Correlate with Notch1 Levels in CCl4 induced liver injury model. Mice were injected with Control or CCl4 (2 ml/kg body weight, i.p., twice a week for 2 weeks). a. Expression of FXR and Notch1 mRNAs in livers of WT and CCl4-treated mice. b Expression of FXR and Notch1 in WT and CCl4-treated mice was examined by western blotting, normalized to GAPDH. Data were presented as mean ± SEM (*N* = 4) of three independent experiments. *P < 0.05; **p < 0.01. **Figure S3.** The BrdU pulse-chase assay analysis in liver cancer cells. a. After two weeks the BrdU pulse, mitotic cells were stained for BrdU labeling by immunofluresence. A representative image is shown in which all of the cells at various degrees of condensed chromatin were BrdU-positive (red). Scale bar: 50 μm. **Figure S4.** FXR activation inhibits Notch1 expression and protects from CCl4 induced liver injury. Liver injury was induced by CCl4 administration (i.p. 2 ml/Kg body weight, twice a week for 2 weeks). CCl4 mice were randomized to receive GW4064 (50 mg/Kg once every two days for 2 weeks) or Control (4:1 of PEG-400 and Tween 80). a serum level of ALT (left) and AST(right) were calculated. b. Representative liver sections from WT or FXR-KO mice stained with H&E. c Quantitative real-time. PCR analysis shown expression of SHP (left) and Notch1 (right), in WT and FXR-KO mice treated as indicated. d Western blotting analysis of NICD1, Notch1 and Numb protein levels in livers of WT and FXR-KO mice, normalized to LaminB or GAPDH. Data represented the mean ± SEM (N = 4). Statistical significance of differences between each treatment and control group (*p < 0.05; **p < 0.01) were determined. **Table S1.** The siRNA-FXR and negative control (Si-NC) sequences. **Table S2.** The primers used for reverse transcription, PCR and qPCR. **Table S3.** The primers used for the expression vector construction. **Table S4.** The EMSA reaction system.

## Data Availability

All data generated or analyzed during this study are included in this article.

## Abbreviations

ACD: Asymmetrical cell division
ALT: Alanine aminotransferase
AST: Aspartate aminotransferase
BrdU: 2-Bromodeoxy-uridine
CDCA: Chenodeoxycholic acid
ChIP: Chromatin immunoprecipitation
CSC: Cancer stem cell
CCl4: Carbon tetrachloride
DAPT: *N*-[*N*-(3,5-difluorophenacetyl)-l-alanyl]-*S*-phenylglycine *t*-butyl ester
DDC: 3,5 Diethoxicarbonyl-1,4 dihydrocollidine
DMEM: Dulbecco’s modified Eagle’s medium
DR: Direct repeat
DMSO: Dimethylsulfoxide
EMSA: Eletrophoretic mobility shift assay
FACS: Fluorescence-activated cell sorting
FBS: Fetal bovine serum
FXR: Farnesoid X receptor
FXRE: Farnesoid X receptor response element
HCC: Hepatocellular carcinoma
IgG: Immunoglobulin G
IP: Intraperitoneal
KO: Knockout
mRNA: Messenger RNA
Mut: Mutant
NICD1: NOTHC1 intracellular domain
PBS: Phosphate-buffered saline
PEG-400: Polyethylene glycol-400
RXR: Retinoid X receptor
SHP: Small heterodimer partner
SCD: Symmetrical cell division
Sox9: SRY (sex-determining region Y)-box 9
SEM: Standard error of the mean
siRNA: Small interfering RNA
WT: Wild type
