# Supplementary material for: Farnesoid X receptor via Notch1 directs asymmetric cell division of Sox9+ cells to prevent the development of liver cancer in a mouse model
Source: Stem Cell Res Ther. 2021 Apr 12;12:232. doi: 10.1186/s13287-021-02298-6 (PMC8042944; doi:10.1186/s13287-021-02298-6)
Supplement: Supplementary file 1 — Additional file 1: Figure S1. Representative photomicrographs of liver lesions from 12 month-old WT and FXR-KO mice. a. Liver tumors in 12-month-oldWT and FXR-KO mice. Circles show the tumor nodules. Figure S2. Hepatic FXR Levels Inversely Correlate with Notch1 Levels in CCl4 induced liver injury model. Mice were injected with Control or CCl4 (2 ml/kg body weight, i.p., twice a week for 2 weeks). a. Expression of FXR and Notch1 mRNAs in livers of WT and CCl4-treated mice. b Expression of FXR and Notch1 in WT and CCl4-treated mice was examined by western blotting, normalized to GAPDH. Data were presented as mean ± SEM (N = 4) of three independent experiments. *P < 0.05; **p < 0.01. Figure S3. The BrdU pulse-chase assay analysis in liver cancer cells. a. After two weeks the BrdU pulse, mitotic cells were stained for BrdU labeling by immunofluresence. A representative image is shown in which all of the cells at various degrees of condensed chromatin were BrdU-positive (red). Scale bar: 50 μm. Figure S4. FXR activation inhibits Notch1 expression and protects from CCl4 induced liver injury. Liver injury was induced by CCl4 administration (i.p. 2 ml/Kg body weight, twice a week for 2 weeks). CCl4 mice were randomized to receive GW4064 (50 mg/Kg once every two days for 2 weeks) or Control (4:1 of PEG-400 and Tween 80). a serum level of ALT (left) and AST(right) were calculated. b. Representative liver sections from WT or FXR-KO mice stained with H&E. c Quantitative real-time. PCR analysis shown expression of SHP (left) and Notch1 (right), in WT and FXR-KO mice treated as indicated. d Western blotting analysis of NICD1, Notch1 and Numb protein levels in livers of WT and FXR-KO mice, normalized to LaminB or GAPDH. Data represented the mean ± SEM (N = 4). Statistical significance of differences between each treatment and control group (*p < 0.05; **p < 0.01) were determined. Table S1. The siRNA-FXR and negative control (Si-NC) sequences. Table S2. The primers used for reverse [file 13287_2021_2298_MOESM1_ESM.pdf]

Supplementary Material

Farnesoid X Receptor via Notch1 directs asymmetric cell division of Sox9$^+$ cells to prevent development of liver cancer in a mouse model

Mi Chen[1], Chenxia Lu[2*], Hanwen Lu[1*], Junyi Zhang[1], Dan Qin[1], Shenghui Liu[1], Xiaodong Li[3] and Lisheng Zhang[1]

[1]College of Veterinary Medicine / College of Biomedicine and Health, Huazhong Agricultural University, Wuhan, 430070, China.

[2]The Clinical Medical College of Traditional Chinese Medicine, Hubei University of Chinese Medicine, Wuhan, 430065, China.

[3]Hubei Provincial Hospital of TCM, Hubei Provincial Academy of TCM. Wuhan 430061, China.

*These authors contributed equally to this work.

Corresponding author:

Lisheng Zhang, College of Veterinary Medicine / College of Biomedicine and Health, Huazhong Agricultural University, Wuhan, 430070, China. Tel: 86-27-87282091, Fax: 86-27-87280470. E-mail: lishengzhang@mail.hzau.edu.cn.

Supplemental Figures

Figure . S1

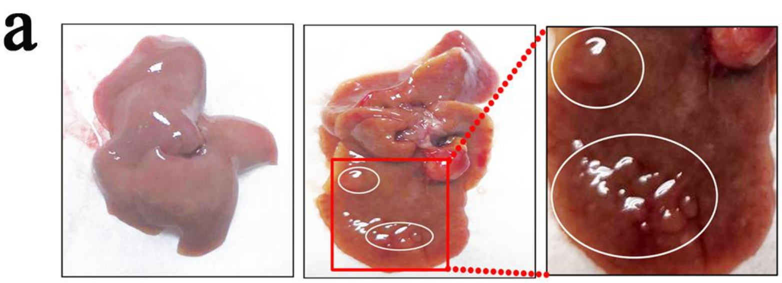

Fig. S1 Representative photomicrographs of liver lesions from 12 month-old WT and FXR-KO mice. **a** Liver tumors in 12-month-old WT and FXR-KO mice. Circles show the tumor nodules.

Figure . S2

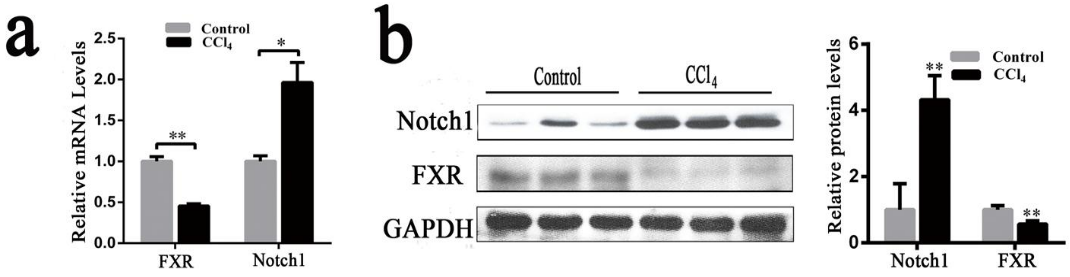

Fig. S2 Hepatic FXR Levels Inversely Correlate with Notch1 Levels in CCl4 induced liver injury model. mice were injected with Control or CCl_4 (2ml/kg body weight, i.p., twice a week for 2 weeks). **a** Expression of FXR and Notch1 mRNAs in livers of WT and CCl_4-treated mice. **b** Expression of FXR and Notch1 in WT and CCl_4-treated mice was examined by western blotting, normalized to GAPDH. Data were presented as mean ± SEM (N = 4) of three independent experiments. $^*P < 0.05$; $^{**}p < 0.01$.

Figure . S3

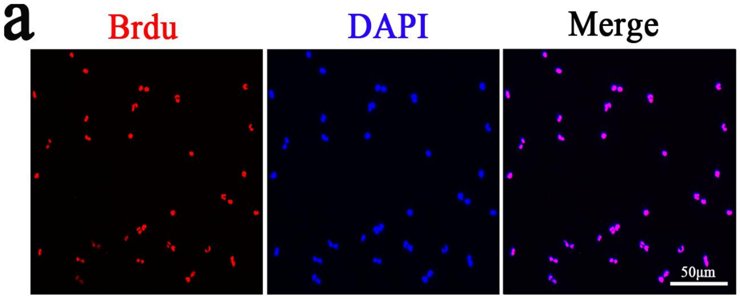

Fig. S3 The BrdU pulse-chase assay analysis in liver cancer cells. **a** After two weeks the BrdU pulse, mitotic cells were stained for BrdU labeling by immunofluresence. A representative image is shown in which all of the cells at various degrees of condensed chromatin were BrdU-positive (red). Scale bar: 50μm.

Figure . S4

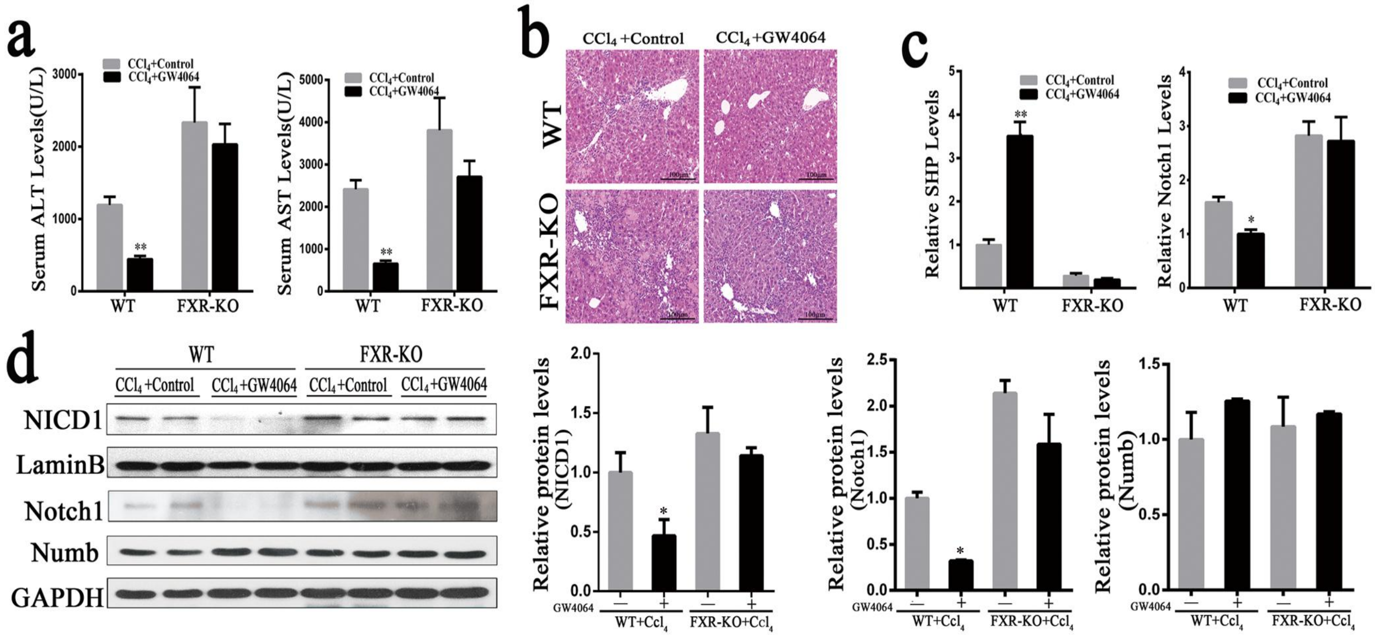

Fig. S4 FXR activation inhibits Notch1 expression and protects from CCl_4 induced liver injury. Liver injury was induced by CCl_4 administration (i.p. 2 ml/Kg body weight, twice a week for 2 weeks). CCl_4 mice were randomized to receive GW4064 (50 mg/Kg once every two days for 2 weeks) or Control (4:1 of PEG-400 and Tween 80). **a** serum level of ALT (left) and AST(right) were calculated. **b**

Representative liver sections from WT or FXR-KO mice stained with H&E. **c** Quantitative real-time PCR analysis shown expression of SHP (left) and Notch1 (right), in WT and FXR-KO mice treated as indicated. **d** Western blotting analysis of NICD1, Notch1 and Numb protein levels in livers of WT and FXR-KO mice, normalized to LaminB or GAPDH. Data represented the mean ± SEM (N = 4). Statistical significance of differences between each treatment and control group (*p < 0.05; **p < 0.01) were determined.

Supplemental Tables

Table S1 The siRNA-FXR and negative control (Si-NC) sequences

siRNA names	Sequences (5'-3')
si-FXR sense	GGAAGAAAGAAUUCGAAAUTT
si-FXR antisense	AUUUCGAAUUCUUUCUUCCTT
negative control sense	UUCUCCGAACGUGUCACGUTT
negative control antisense	ACGUGACACGUUCGGAGAATT

Table S2 The primers used for reverse transcription, PCR and qPCR

Primer names	Sequences (5'-3')
H-GAPDH-Forward	CTCTGGTAAAGTGGATATTG
H-GAPDH-Reverse	CTCTGGTAAAGTGGATATTG
H-FXR-Forward	ATGCCTGTAACAAAGAAGCCCC
H-FXR-Reverse	CACACAGTTGCCCCCGTTTTTA
H-SHP-Forward	GTCCAGCTATGTGCACCTCATC
H-SHP-Reverse	TTCCTGAGGAAGGCCACTGT
H-Notch1-Forward	GTCAACGCCGTAGATGACC
H-Notch1-Reverse	TTGTTAGCCCCGTTCTTCAG
H-Bsep-Forward	GGAGCATTGACAACAAGACT
H-Bsep-Reverse	CATTTGTAATCTGTCCCACC
Chip-Notch1-Forward	TGCCTGGCTGCTGTTACATAA
Chip-Notch1-Reverse	ATAATCTGGCCTCACTTCTGC
m-36B4-Forward	TGGAGACAAGGTGGGAGCC
m-36B4-Reverse	CACAGACAATGCCAGGACGC
m-SHP-Forward	CCTCTACCCTCAAGAACATTCCA
m-SHP-Reverse	TTCAGTGATGTCAACGTCTCCC
m-Notch1-Forward	TGAATGGAGGGAGGTGCGAAGT
m-Notch1-Reverse	GTGCTGAGGCAAGGATTGGAGT
m-FXR-Forward	TCCGGACATTCAACCATCAC
m-FXR-Reverse	TCACTGCACATCCCAGATCTC

Table S3 The primers used for the expression vector construction

Primer names	Sequences (5'-3')
H-Notch1-OE-Forward	GGGGTACCATGCACCTGGATGCCGCTGACCTG
H-Notch1-OE-Reverse	CCCTCGAGCTTGAAGGCCTCCGGAATGCG
pGL3-Notch1 FXRE-wt Forward	GGGGTACCGGCAGTCGCACCCGCACCCGATCAGCA
pGL3-Notch1 FXRE-wt Reverse	GCAAGCTTAGCGCGGGCAGCAGCGCCAGGCAGAGC
pGL3-Notch1 FXRE-mut Forward	GCGCGTCAAACAAGATGTTACCCCAGG
pGL3-Notch1 FXRE-mut Reverse	CCTGGGGTAACATCTTGTTTGACGCGC

Table S4 The EMSA reaction system

	NC reaction	Sample reaction	Cold competitor	Mutant cold competitor	Super-Shift
Nuclease-Free Water	7	5	1	1	4
5×binding buffer	2	2	2	2	2
nuclear protein	0	2	2	2	2
Labeled probe	1	1	1	1	1
Unlabeled probe	0	0	4	0	0
Unlabeled mutated probe	0	0	0	4	0
FXR Antibody	0	0	0	0	1
Total volume(μl)	10	10	10	10	10
